# Correction: Brain–Computer Interface–Based Communication in the Completely Locked-In State

**DOI:** 10.1371/journal.pbio.1002629

**Published:** 2018-04-25

**Authors:** The PLOS Biology Staff

The wrong Academic Editor name and affiliation was included on the original published article. The publisher apologizes for the error. The correct Academic Editor name and affiliation is: Karunesh Ganguly, University of California, San Francisco, UNITED STATES.

## References

[1] Chaudhary U, Xia B, Silvoni S, Cohen LG, Birbaumer N (2017) Brain–Computer Interface–Based Communication in the Completely Locked-In State. PLoS Biol 15(1): e1002593 https://doi.org/10.1371/journal.pbio.1002593 28141803 10.1371/journal.pbio.1002593 PMC5283652

